# The complete mitochondrial genome sequence of *Acisoma panorpoides* Rambur, 1842 (Odonate: Libellulidae)

**DOI:** 10.1080/23802359.2019.1678416

**Published:** 2019-10-18

**Authors:** Lingzhen Cao, Wen Hou, Chaoxing Hu

**Affiliations:** a College of Life Science, Jiangxi Normal University, Nanchang, China;; b College of Agriculture, Guizhou University, Guiyang, China

**Keywords:** Odonata, Acisoma, taxonomy, mitochondrial genome, phylogeny

## Abstract

The phylogenetic relationships of dragonflies have received great attention all the time. For a better understanding the phylogenies among odonate insects, the paper presented the complete mitochondrial genome of *Acisoma panorpoides* based on next generation sequencing data of total genomic DNA. The total length comprised 15,249 bp and the 37 genes (2 rRNA genes, 13 protein coding genes and 22 tRNA genes). Gene content and gene arrangement were identical to other odonate mitogenomes. Phylogenetic analyses using the whole sequences of the mitochondrial genome placed *A. panorpoides* as a sister species to *Hydrobasileus croceus* in Libellulidae.

The Odonata is consisted of three suborders (Zygoptera, Anisozygoptera and Anisoptera) with approximately 6500 described species. Odonate insects play a key role in the evolution of winged insects and as environmental indicators (Catling 2005). The phylogenetic relationships among families of Odonata (Dijkstra et al. 2014; Carle et al. 2015) have traditionally been controversial. In this paper, we sequenced and annotated the complete mitogenome sequences of *Acisoma panorpoides* Rambur, 1842 (Odonata: Libellulidae) which will provide the help in understanding the diversity and phylogeny of dragonflies in the future.

The adults of *Acisoma panorpoides* were collected from Nanchang city of Jiangxi province (28°66′87″N, 115°97′93″E), China, in July 2018. The specimen (ODA-1） was preserved in the herbarium of Jiangxi Normal University, Nanchang, China. The total genomic DNA was extracted from the flight muscles of a single individual using the TIANamp Genomic DNA Kit (Tiangen Biotech, Beijing, China) following the manufacturer’s protocol. The whole mitochondrial genome sequencing was performed in Shanghai Majorbio Bio-Pharm Technology Co., Ltd (Shanghai, China). The mitochondrial genome was assembled using SOAPdenovo v2.04 (http://soap.genomics.org.cn/) and annotated via available mitochondrial genomes of other odonate species (Lorenzo-Carballa et al. 2014; Yu et al. 2014; Feindt et al. 2016; Herzog et al. 2016). The sequences of the other 18 odonate mitochondrial genomes and *Parafronurus youi* were downloaded from GenBank and were used to construct the minimum evolutionary tree (10000 replicates) using MEGA X (Kumar et al. 2018).

The complete mitochondrial genome sequence of *A. panorpoides* (GenBank accession no. MN046207) was a typical circular DNA molecule with the length of 15,249 bp. *Acisoma panorpoides* encoded an A + T-rich control region and a typical set of 37 mitochondrial genes, including 13 protein-coding genes, 2 rRNA genes, 22 tRNA genes. The arrangement and orientation of the mitochondrial genes are identical to the other odonate mitochondrial genomes (Simon and Hadrys 2013; Wang et al. 2015; Feindt et al. 2016). The base composition of the mitochondrial genome is A: 34.6%, T: 39.8%, C: 11.1% and G: 14.5%, with an A + T content of 74.4%. All protein coding genes employed the standard start codon for invertebrate mitochondrial genome: two use TTG (*cox1* and *nad4L*); three use ATT (*atp8, nad5,* and *nad6*); three use ATA (*nad1, nad2*, and *nad3*) and five genes start ATG (atp6, cox3, nad4, cob and cox2). The standard stop codon TAA was used 12 times (*nad2, cox1, cox2, cox3, atp8, atp6, nad4L, nad6, nad5, cob, nad1,* and *nad2*), except TAG was used only once by nad3. The length of 22 tRNA ranged from 64 bp to 71 bp and all tRNA genes have the typical cloverleaf structure except for trnS1.

In the mitogenome of *A. panorpoides*, there were six non-coding intergenic regions including S5 (*nad1/trnL1*) which often lack in Zygopterans (Herzog et al. 2016; Kim et al. 2018). The phylogenetic tree supported *A. panorpoides* as a sister species to *Hydrobasileus croceus* (Figure 1). To better understand the odonate phylogeny on multiple taxonomic levels better, it is necessary to increase gradually dragonfly taxon sampling and sequencing.

**Figure 1.**
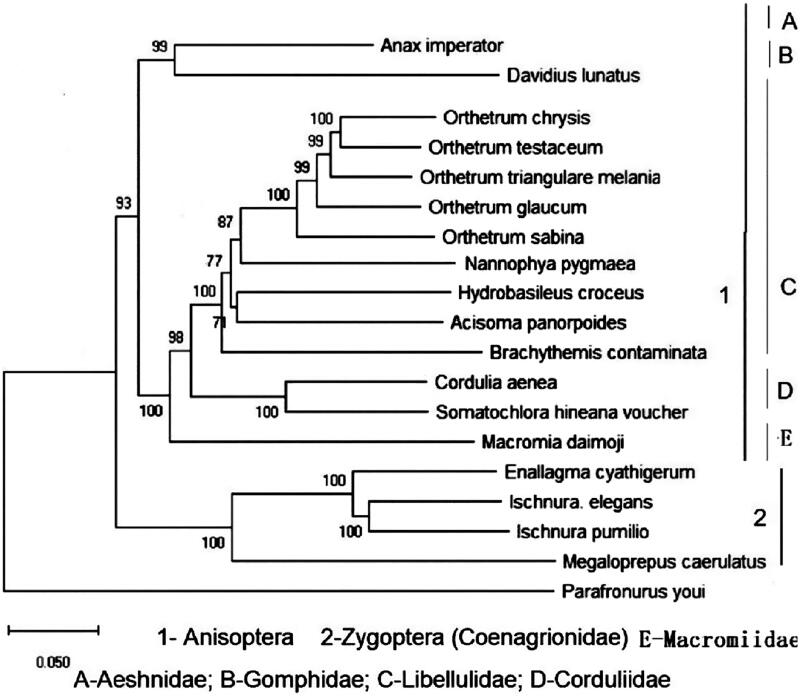
Mitochondrial phylogenetic relationships of 18 Odonata species obtained using complete sequences. GenBank accession numbers are as follows: *Anax imperator* (KX161841); *Davidius. lunatus* (EU591677); *Orthetrum chrysis* (KU361233); *Orthetrum testaceum* (KU361235); *Orthetrum triangulare melania* (AB126005.1); *Orthetrum glaucum* (KU361232); *Orthetrum Sabina* (KU361234); *Nannophya. pygmaea* (KY402222); *Hydrobasileus croceus* (KM244659); *Brachythemis contaminate* (KM658172); *Cordullia aenea* (JX963627); *Somatochlora hineana* voucher (MG594801.1); *Macromia daim*oji (MF990748); *Enallagma cyathigerum* (MF716899); *Ischnura elegans* (KU958378); *Ischnura pumilio* (KC878732); *Megaloprepus caerulatus* (KU958377); *Parafronurus youi* (EU349015.1).

## Geolocation information

The species were collected in Jiangxi Normal University (28°66′87″N, 115°97′93″E), Nanchang city of Jiangxi province, China. The species were preserved in the herbarium of College of Life Science, Jiangxi Normal University, Nanchang, China.
